# An Electrochemical Nucleic Acid Biosensor for Triple-Negative Breast Cancer Biomarker Detection

**DOI:** 10.3390/s24175747

**Published:** 2024-09-04

**Authors:** Lexi Hansen, Sanket Naresh Nagdeve, Baviththira Suganthan, Ramaraja P. Ramasamy

**Affiliations:** Nano Electrochemistry Laboratory, College of Engineering, University of Georgia, Athens, GA 30602, USA; alexa.hansen@uga.edu (L.H.); sanket.nagdeve@uga.edu (S.N.N.); baviththira.suganthan@uga.edu (B.S.)

**Keywords:** miRNA-10b, electrochemical impedance spectroscopy, early diagnosis, quantitative method, human serum

## Abstract

Triple-negative breast cancer (TNBC) is the most aggressive subtype of breast cancer, affecting younger women and women of minorities. The nomenclature “triple negative” is derived from the absence of the three most common breast cancer biomarkers: progesterone receptor (PR), estrogen receptor (ER), and human epidermal growth factor receptor 2 (HER2). It derives its name from testing negative for these three most common breast cancer biomarkers. Currently, TNBC is diagnosed at advanced stages, necessitating the need for a diagnostic tool or method to identify this malignancy at an early stage prior to metastasis. In this study, a novel electrochemical biosensor was developed, optimized, and evaluated for the detection of microRNA-10b (miRNA-10b), marking the first use of this biomarker for the early diagnosis of TNBC. The biosensor demonstrated the ability to detect concentrations as low as 10 pM. Furthermore, the biosensor was specific toward the target biomarker, distinguishing non-target miRNAs of similar size. The efficacy of the biosensor for TNBC early diagnosis was further validated using human serum samples.

## 1. Introduction

Breast cancer is the most commonly diagnosed cancer among women [1]. Different subtypes of breast cancer are classified based on the expression of three common biomarkers: progesterone receptor (PR), estrogen receptor (ER), and human epidermal growth factor receptor 2 (HER2). The most common four subtypes are HER2+, Luminal A, Luminal B, and Triple-negative breast cancer (TNBC) [2]. TNBC accounts for 10–20% of all cancer diagnoses. TNBC-affected individuals test negative for ER, PR, and HER2, hence the name for this subtype. TNBC is known for its aggressiveness, poor survival rate, and higher rate of reoccurrence [3,4]. TNBC disproportionally affects younger women, women of African American descent, and women with Breast Cancer gene 1 (BRCA 1) mutation diagnoses [5,6,7,8,9]. The current diagnostic method takes multiple steps, is expensive, and does not diagnose TNBC promptly. TNBC is currently diagnosed in higher stages, with 12% of women diagnosed at stage 1, 11% diagnosed at stage 2, 52% diagnosed at stage 3, and 25% diagnosed at stage 4 [10]. The majority of TNBC patients are diagnosed once the cancer has metastasized to other parts of the body, which limits treatment options and decreases the chance of survival. There is an unmet need for a new detection method that can accurately detect TNBC at an earlier stage than currently afforded, allowing for increased treatment options and a better chance of survival. Exosomes, circulating tumor cells, and microRNAs (miRNAs) are recognized as novel biomarker sources for metastatic breast cancer [11]. miRNAs are considered more effective biomarkers than exosomes and circulating tumor cells for identifying TNBC at an early stage due to their stability in blood, which ensures consistent and reliable detection over time [12]. They can be detected at very low concentrations, whereas circulating tumor cells may be undetectable. In addition, the dysregulation of specific miRNAs provides high specificity, thereby improving the accuracy of early diagnosis [13].

miRNAs are small noncoding ribonucleic acid (RNA) sequences involved in various biological processes, such as differentiation, proliferation, apoptosis, and regulation [14,15,16,17,18,19,20]. miRNAs can regulate messenger RNAs, which control protein expression through translation [21]. miRNAs are tightly regulated; therefore, dysregulation of any miRNAs, whether upregulation or downregulation, can be associated with numerous disorders, including TNBC [22,23]. Studies have identified over 30 miRNAs involved in TNBC and reported miRNA-10b, miRNA-9, and miRNA-17-5P as three potential biomarkers for TNBC [24,25,26,27]. Kim et al. completed an in vivo mice study showing that the removal of miRNA-10b, when compared to mice who still had miRNA-10b, caused a 73% reduction in tumor size [28]. Studies have linked high levels of miRNA-10b to metastatic breast cancer, with an increase of 5- to 6-fold compared to benign and primary breast cancers [29]. The current literature reports miRNA expression levels as a ratio compared to a standard level using quantitative reverse transcriptase polymerase chain reaction (qRT-PCR) instead of reporting the concentration of the detected miRNAs [30,31,32]. 

Electrochemical biosensors provide several key advantages for detecting miRNAs, including low detection limits and minimal equipment cost. In addition, they allow for the simultaneous detection of multiple analytes and require minimal sample volumes. These advantages make electrochemical biosensors an efficient and effective tool for detecting miRNAs [33,34]. The literature reports several electrochemical biosensors that detect miRNAs using DNA immobilization techniques [35,36,37,38,39,40,41]. However, there are no established biosensors for detecting miRNA-10b using DNA immobilization techniques.

In this work, a new method was developed to detect the biomarker miRNA-10b using an electrochemical biosensing platform and tested as a potential new tool for early TNBC diagnosis. The literature has demonstrated the use of electrochemical biosensors for detecting breast cancer biomarkers, such as miRNA-21 and miRNA-155 [42]. However, the application of miRNA-10b as a potential biomarker for detecting TNBC has not been reported yet.

The development of a biosensor involves several critical steps to enhance its effectiveness. Carbon-based graphene is utilized as a nanomaterial to improve the electrical conductivity and increase the surface area of the electrode [43]. 1-pyrene butanoic acid succinimidyl ester (PBSE) is used as a crosslinker to attach single-stranded deoxyribonucleic acid (ssDNA) to the graphene-modified electrode surface [44,45]. PBSE allows for the consistent and stable functionalization of graphene, facilitated through strong π–π stacking interactions with the graphene surface, thereby enhancing the reliability and reproducibility of the biosensor signals. Furthermore, PBSE is compatible with both organic solvents and aqueous environments, making it suitable for various biosensing applications. 

In this work, we present a novel electrochemical biosensor that uses a nanostructured electrode as a transducer for the detection of miRNA-10b. This biosensor is able to detect miRNA-10b using an ssDNA probe as a bioreceptor, which was functionalized on the transducer surface. The amount of the target analyte (miRNA-10b) in the sample was quantified via an impedimetric measurement that measures the charge transfer resistance (*R_CT_*) of an electrochemical reaction at the transducer surface. The sensitivity of the optimized biosensor was evaluated according to the various concentrations of miRNA-10b. The specificity of the biosensor was analyzed by using non-complementary miRNAs as pseudo-analytes. Finally, the biosensor was evaluated for its ability to detect miRNAs in serum samples. 

## 2. Materials and Methods

### 2.1. Materials

Graphene (X and Y dimensions: 1–2 μm, grade 4, purity: >99 wt.%) was obtained from Cheap Tubes Inc., Cambridgeport, VT, USA; dimethylformamide (DMF) from Acros Antwerpen, Belgium; potassium ferricyanide (K_3_[Fe(CN)_6_]) from AMRESCO Inc. Solon, OH, USA; potassium ferrocyanide (K_4_[Fe(CN)_6_]) from Fischer Chemicals Pittsburgh, PA, USA; PBSE from AnaSpec, Fremont, CA, USA; sodium phosphate monobasic anhydrous (NaH_2_PO_4_) from Research Products International Corps Mt. Prospect, IL, USA and sodium phosphate dibasic anhydrous (Na_2_HPO_4_) from EMD Millipore Burlington, MA, USA; magnesium chloride hexahydrate (MgCl_2_.6H_2_O) from MP Biomedicals Santa Ana, CA, USA; sodium chloride (NaCl) from Fischer Chemicals Pittsburgh, PA, United States; potassium chloride (KCl) from VWR Chemicals, Solon, OH, USA; sterile-filtered human serum (human AB plasma) and RNaseZap from Sigma-Aldrich, St. Louis, MO, USA. Alumina powder type N of size 0.05 µm was obtained from Electron Microscopy Sciences, Hatfield, PA, USA. The miRNA and deoxyribonucleic acid (DNA) oligonucleotides were purchased from Eurofins Genomics, Louisville, KY, USA. The miRNA-10b strands used for optimization and specificity studies are listed below.

*miRNA-10b: 5′-UAC CCU GUA GAA CCG AAU UUG UG-3′;*
*Anti-miRNA-10b (DNA probe strand): 5′-NH_2_-C_6_-CAC AAA TTC GGT TCT ACA GGG TA-3′;*
*Non-Target miRNA 1: 5′-GGC CCA CUA GCA CCU AAC UGG UA-3′;*
*Non-Target miRNA 2: 5′-UAG ACU GUA CAA CUG ACU UUG GG-3′.*

Stock solutions of the oligonucleotides (10 µM) were prepared using molecular biology grade water from Corning, Tewksbury MA, USA and kept at −80 °C. Smaller aliquots in needed concentrations (1 µM–1 fM) were prepared to avoid repeated thaw/freeze cycles and stored at −80 °C. 

The following buffers were prepared using molecular grade water and used for this experiment: phosphate buffer (PB): 10 mM Na_2_HPO_4_/NaH_2_PO_4_, a hybridization buffer: 10 mM PB + 1 M NaCl + 20 mM MgCl_2_.6H_2_O, and an immobilization buffer: PB + 0.15 M NaCl. 

RNAaseZap solution was used to decontaminate all of the surfaces, tubes, and glass containers that may come into contact with RNA. Disposable RNAse, DNAse, and protease-free filter pipette tips and microtubes were used for all of the experiments. Separate pipettes were used for DNA and RNA-related work. In addition, hair nets were worn to ensure no strands of hair particles contaminated the workspace. 

### 2.2. Methods

#### 2.2.1. Fabrication of the ssDNA-Immobilized Electrode

The glassy carbon electrode (GCE) was polished using a 0.05 μm alumina powder slurry on a polishing pad for 5 min. The GCE was rinsed with deionized water and cleaned using a bath sonicator for 5 min. Once again, the electrode was rinsed using deionized water to remove all alumina particles and placed into an oven at 70 °C to dry for 45 min. Then, the electrode was allowed to cool to room temperature before graphene was deposited. The graphene mixture was created using 2 mg of graphene in 1 mL of DMF. This solution was probe-sonicated for an hour. Two microliters of the graphene solution were deposited on the GCE working surface and were allowed to dry uniformly for 1 h at room temperature. Two microliters of 10 mM PBSE were added to the GCE and incubated in ice for 15 min to allow the aromatic rings on PBSE to bind to the graphene. To ensure no unbound PBSE molecules were left, the GCE was washed with DMF and then washed with 0.01 M PB twice to ensure all DMF was removed from the surface of the electrode for the immobilization of the ssDNA. 

The electrode was washed with the immobilization buffer, and 30 µL of ssDNA solution (2 µM in immobilization buffer) was deposited. The electrode was incubated for an hour at room temperature in a glass desiccator. The modified electrode was washed with PB to remove any excess ssDNA molecules that did not bind to the surface. A blocking agent ethanolamine (10 mM in PB) was dropped on the electrode and incubated for 30 min to inactivate any unreacted PBSE molecules. The electrode was washed with PB twice to ensure all ethanolamine was removed, and the baseline reading was taken using Electrochemical Impedance Spectroscopy (EIS). 

#### 2.2.2. Hybridization of miRNA-10b

Before depositing the target miRNA-10b on the surface, the miRNA was heated for 5 min at 80 °C and allowed to cool to room temperature. This allowed all of the miRNA to unfold and enabled hybridization. Thirty microliters of miRNA-10b were pipetted onto the fabricated GCE surface and incubated at room temperature for an hour before being washed with the hybridization buffer. It was washed with PB twice to ensure all unbound RNA molecules were removed. EIS measurements were taken.

#### 2.2.3. Electrochemical Characterization

All experiments were conducted using Gamry Instruments Interface 1010E, Warminster, PA, USA. EIS was run with an alternating current (AC) of 5 mV between frequencies of 1 Hz and 10 kHz. Characteristics analyzed by the EIS readings included Δ*R_CT_*, defined as follows:

$$\Delta R_{CT}=R_{CT}(after\;hybridization)-R_{CT}(before\;hybridization)$$

The % increase in *R_CT_* was also analyzed using the formula below:

$$\%\;\;Increase\;R_{CT}=\frac{R_{CT}\;\left(after\;hybridization\right)-R_{CT}\left(before\;hybridization\right)}{R_{CT}\left(before\;hybridization\right)}\times 100$$

The limit of detection (LOD), limit of quantification (LOQ), and sensitivity are described below, where σ = the standard deviation of the response and S = the slope of the calibration curve.

$$LOD=3.3*\left(\frac{\sigma }{S}\right)$$

$$LOQ=10*\left(\frac{\sigma }{S}\right)$$

$$Sensitivity=\frac{Slope\;of\;the\;calibration\;curve}{Surface\;area\;of\;the\;working\;electrode}$$

EIS was run using a three-electrode system consisting of a working, counter, and reference electrode. All three were purchased from CH Instruments Inc., Austin, TX, USA. The working electrode (glassy carbon electrode), reference electrode (3 M Ag/AgCl), and counter electrode (platinum wire) were placed in a 0.1 M KCl electrolyte solution containing 5 mM Fe (CN)_6_ ^3−/4−^ (1:1). 

#### 2.2.4. Biosensor Testing and Validation

Once the optimization of the electrode fabrication was finalized, the biosensor was tested and validated. To determine the sensitivity, the biosensor was tested over a range of concentrations (1 μM to 100 pM) to produce a calibration curve. Two different non-complementary miRNAs with 23 base pairs were used to determine the specificity of the biosensor. When evaluated in buffer solutions, a biosensor can demonstrate a certain degree of sensitivity and specificity; nevertheless, testing with human samples is required before it can be employed in practical applications. The literature shows that miRNA-10b levels are elevated in human serum [30,46,47,48]. The biosensor’s ability to detect miRNA-10b was assessed by spiking a two-fold diluted serum sample before depositing the sample on the surface of the biosensor for the hybridization period [49,50,51,52,53]. After the DNA immobilization and ethanolamine steps, the diluted serum (no miRNA-10b) was heated up at 80 °C for five minutes before being deposited on the surface of the electrode for an hour to minimize the detection of non-specific adsorption [54]. The diluted serum was spiked and heated at 80 °C for five minutes before being placed on the electrode for an hour for hybridization. The stability of the biosensor can determine the potential shelf life for a point-of-care device. The electrode was modified with PBSE and incubated in 1 mM PB at 4 °C for the stability testing experiment. To evaluate the stability, the fabricated biosensor was stored in 1 mM PB at 4 °C for 10 days, and the response was measured at three different time stability time points: 3, 7, and 10 days. At the end of each time frame, the biosensor was removed from the incubation, and EIS measurements were taken for a new DNA baseline before hybridizing the electrode with 1 μM of RNA.

## 3. Results and Discussion

### 3.1. The Fabrication and Working Principle of the Biosensor

**Figure 1:**
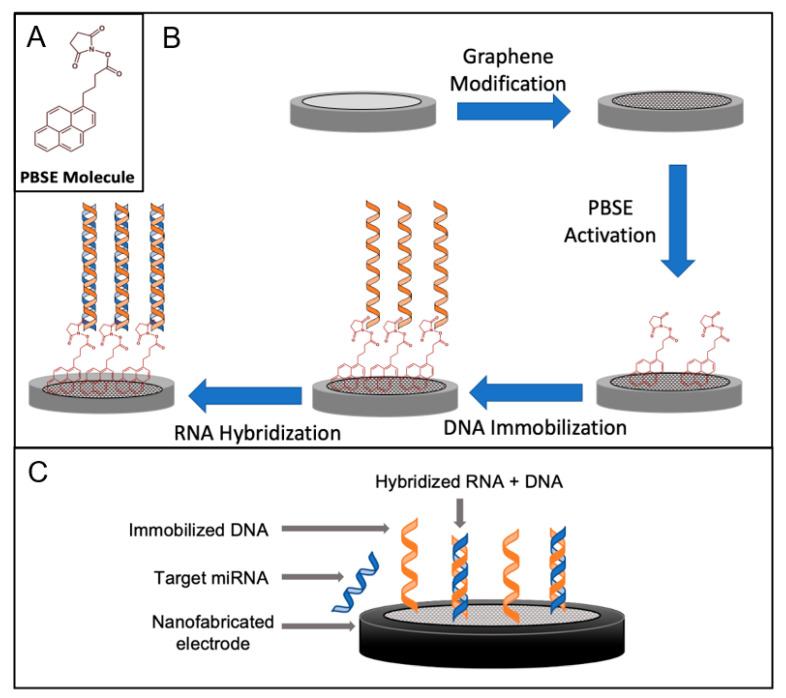
PBSE molecule (**A**), the individual steps for a fabricated graphene-modified nucleic acid biosensor for miRNA detection (**B**), and the fully fabricated biosensor (**C**).

**Figure 2:**
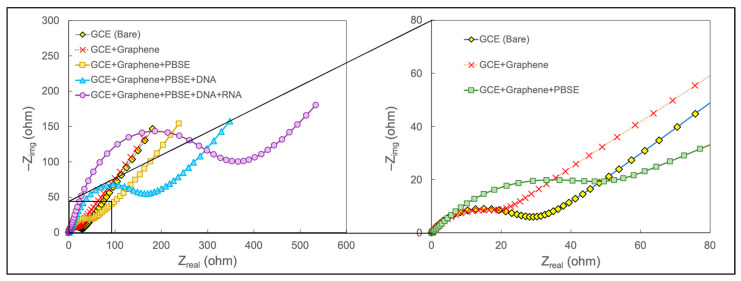
The impedance response for different modifications on the glassy carbon electrode.

The fabrication of the biosensor is shown in Figure 1. Graphene was added to a glassy carbon electrode, and the nanostructured electrode was prepared. Graphene is used in electrochemical biosensors due to its exceptional electrical, mechanical, and chemical properties [43]. Its large surface area, high electrical conductivity, and biocompatibility make it an ideal platform for immobilizing biomolecules and enhancing electron transfer kinetics, leading to the sensitive and rapid detection of biomarkers. Probe DNA molecules were attached to the surface using PBSE as the crosslinker. PBSE acts as a crosslinker, facilitating the immobilization of biomolecules onto the graphene surface via π–π bonding between the aromatic pyrene rings of PBSE and the plane of the graphene [55]. The amino group on the end of the DNA molecule reacts with the ester group of PBSE, forming a covalent amide bond and successfully immobilizing the ssDNA probe onto the surface. The complementary nucleotides between the ssDNA and the target miRNA allow for hybridization, increasing the surface impedance. The signal between different layers of the biosensor was observed using a Nyquist plot, as shown in Figure 2.

### 3.2. Optimization of Experimental Conditions

**Figure 3:**
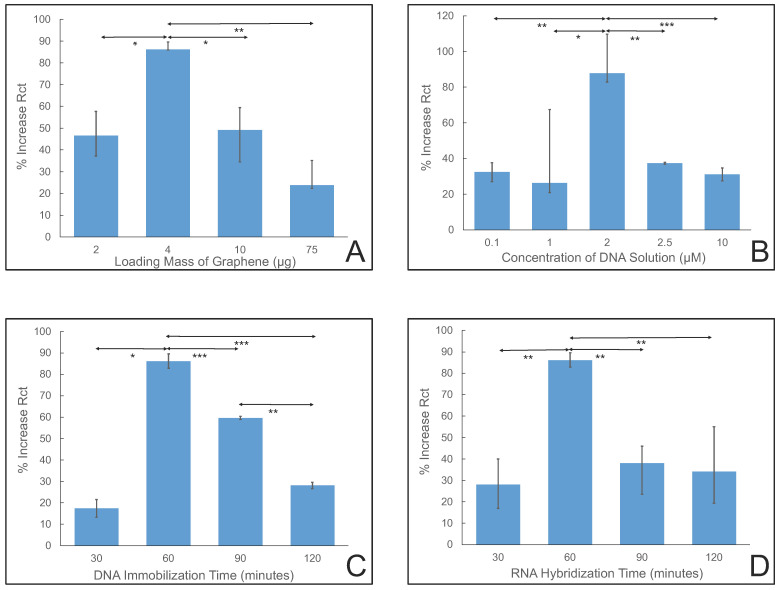
Impedance response for different optimization steps on glassy carbon electrode. Graphene loading and its effect on % increase in *R_CT_* (**A**). DNA concentration and its effect on % increase in *R_CT_* (**B**). DNA immobilization time and its impact on % increase in *R_CT_* (**C**). RNA hybridization time and its effect on % increase in *R_CT_* (**D**). The asterisk indicates statistical significance (* *p* < 0.05, ** *p* < 0.01 and *** *p* < 0.001 using a two-tailed test).

Several variables were optimized, including the mass of the graphene, concentration of the DNA, immobilization time of the DNA, and hybridization time of the RNA, to obtain the highest response of the biosensor with the shortest detection time. It is crucial to consider graphene loading for an electrochemical biosensor. By analyzing the loadings tested (2 μg, 4 μg, 10 μg, and 75 μg of graphene), a balanced approach was chosen to ensure reliable detection without compromising sensitivity or causing interference. As shown in Figure 3A, low loading (2 μg) was deemed inadequate due to insufficient coverage of the sensor’s surface, potentially leading to reduced sensitivity. High concentrations, such as 10 μg and 75 μg, mean densely packed graphene layers, which could obstruct electron transfer and make potential binding sites less accessible. As a result, 4 μg graphene loading was selected to balance sensitivity and coverage, giving enough surface area for effective miRNA-10b binding without resulting in aggregation or interference [56]. This approach aims to maximize detection accuracy while minimizing potential drawbacks associated with extreme graphene loading, ensuring the biosensor’s optimal performance in miRNA-10b detection applications.

It is essential to consider various DNA concentrations and their effects on sensor performance to optimize DNA concentration for the designed biosensor. Five different DNA concentrations, 0.1 μM, 1 μM, 2 μM, 2.5 μM, and 10 μM, were analyzed, and the signal from the sensor was observed. Figure 3B shows that for low concentrations, including 0.1 μM and 1 μM, the biosensor resulted in a weak hybridization signal, whereas high concentrations, such as 2.5 μM and 10 μM, overcrowded the DNA molecules on the sensor’s surface, causing steric hindrance and reduced sensitivity [57]. Hence, the optimal concentration observed was 2 μM, which offered adequate signal strength, reduced extra noise, and provided surface coverage for effective miRNA-10b detection. This concentration ensured reliable detection without compromising sensor performance.

The effect of DNA immobilization time was studied to determine its impact on the biosensor’s performance. Various immobilization times were chosen, including 30 min, 60 min, 90 min, and 120 min. Figure 3C shows that a balance between efficiency and effectiveness was observed for 60 min of immobilization time. Shorter immobilization times do not provide sufficient opportunity for DNA binding to the PBSE molecule, potentially leading to incomplete target recognition and lower detection sensitivity. However, a long immobilization time (120 min) could result in non-specific binding and background noise, compromising the biosensor’s specificity and increasing the risk of false positives [58]. Therefore, the optimal immobilization duration is around or less than 60 min, allowing for robust probe DNA hybridization while minimizing non-specific interactions, thus maximizing the biosensor’s performance in miRNA-10b detection applications.

Optimizing the hybridization time for the electrochemical biosensor is crucial for enhancing its performance. Four different time durations were chosen to explore the impact of the time intervals on the sensor’s efficiency, namely 30 min, 60 min, 90 min, and 120 min. As shown in Figure 3D, a shorter hybridization time, like 30 min, leads to insufficient binding between the miRNA and the ssDNA molecule, resulting in decreased accuracy and sensitivity [59]. However, a long hybridization time (120 min) can lead to non-specific binding, increased background noise, and potential degradation of the sensor’s components, ultimately compromising its reliability and specificity. Therefore, striking a balance between efficient hybridization and minimizing non-specific binding is essential. Based on the findings, a hybridization time of around 60 min is optimal, ensuring sufficient binding while mitigating potential drawbacks associated with shorter or longer durations. This optimized hybridization time can enhance the electrochemical biosensor’s sensitivity, specificity, and performance in miRNA-10b detection.

### 3.3. The Sensitivity of the Biosensor

**Figure 4:**
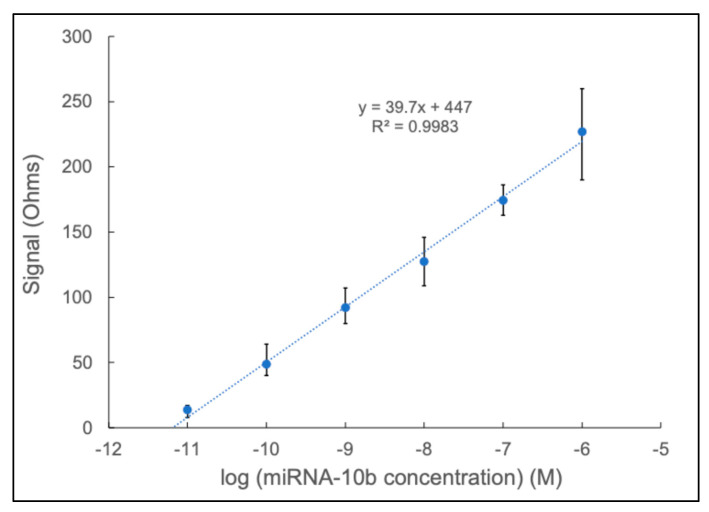
The buffer calibration curve for miRNA-10b over a range from 1 μM to 10 pM.

Figure 4 showcases the calibration curve based on the Δ*R_CT_* averages over six concentrations. The higher concentrations could have high variability due to reaching the saturation limit and compromising the surface of the biosensor, which decreases the available places for a redox reaction to occur [53,54]. As anticipated in a direct hybridization reaction, the change in values followed a linear pattern (correlation factor R^2^ = 0.998) as the concentration of miRNA-10b ranged from 10^−6^ M to 10^−11^ M. Based on the data, the calculated sensitivity value is 55,915.5 Ω dm mol^−1^. The LOD refers to the concentration of the measured substance, where the signal noticeably differs from the background noise. The resulting LOD was determined to be 10^−11^ M for the developed biosensor. 

### 3.4. The Specificity of the Biosensor

**Figure 5:**
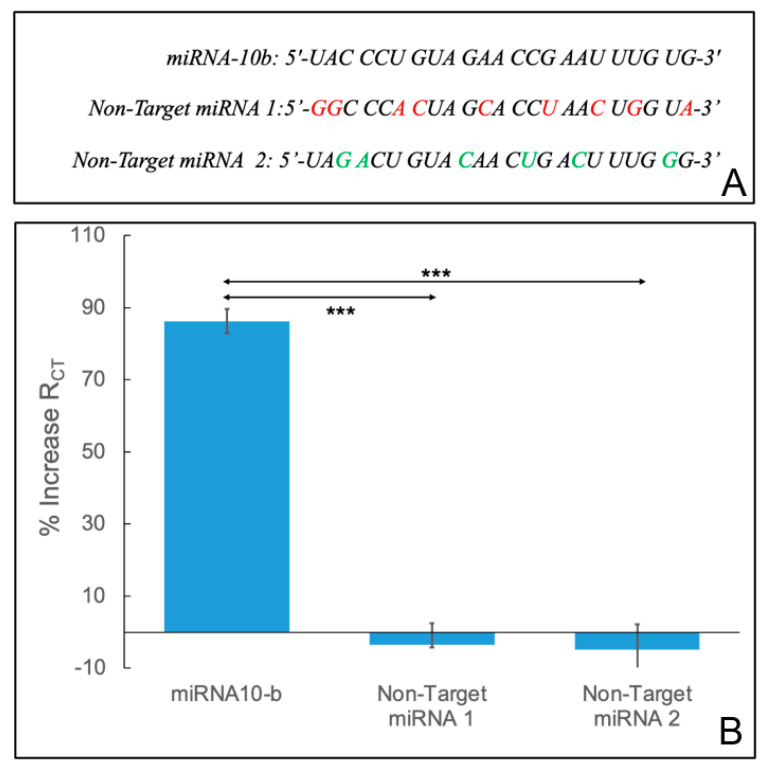
The miRNA-10b sequence compared to the two non-target sequences (**A**) and the biosensor’s response to the target miRNA-10b and non-target miRNAs (**B**). The asterisk indicates statistical significance (*** *p* < 0.001 using a two-tailed test).

The specificity of the developed biosensor was evaluated using two non-complementary miRNA sequences, as shown in Figure 5A, which were used as non-targets (non-target miRNA 1 and non-target miRNA 2) and subsequently hybridized with the DNA-immobilized electrode. The signals were obtained and compared to the complementary miRNA-10b. The specificity of the biosensor was analyzed in two conditions: buffer and serum. As shown in Figure 5B, a significant *R_CT_* drop was observed for the two non-targets. This is due to the fact that ssDNA is notably less stable than double-stranded DNA (dsDNA) or ssDNA–single-stranded RNA (ssRNA) hybrid duplexes. This reduced *R_CT_* is attributed to the thermodynamically less stable nature of ssDNA structures, making them more susceptible to degradation and conformational changes [60,61]. Therefore, the developed electrochemical miRNA sensor demonstrates exceptional specificity and a remarkable ability to differentiate between similar but non-complementary miRNA sequences. However, the results can be improved by reducing the non-specific bonds occurring on the electrode’s surface. 

### 3.5. The Stability of the Biosensor

Understanding the stability of the biosensor is crucial to ensure it provides reliable and consistent behavior. The stability experiments were carried out using two different methods. In the first method, the DNA-immobilized biosensor was evaluated for stability over 0, 3, 7, and 10 days. The biosensor was stored in an immobilization buffer at 4 °C. Once removed, the miRNA was added and allowed to hybridize. As shown in Supplementary Figure S1, the biosensor was able to retain its signal at 73% on the 3rd day of the study. After 7 days, the biosensor’s stability drastically dropped to 50% since the single-stranded DNA is not stable compared to the dsDNA or ssDNA-ssRNA hybrid duplex [60,61]. 

**Figure 6:**
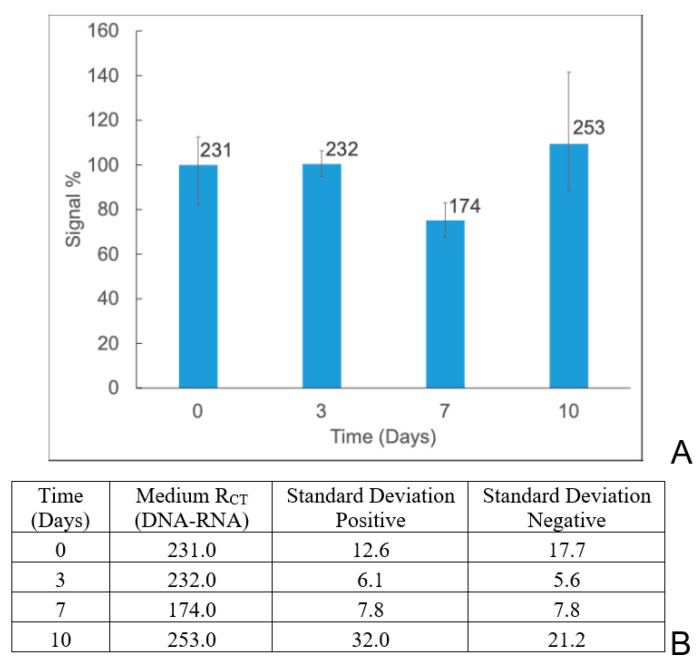
Time stability study over 10 days (**A**), and the table represents the actual values of *R_CT_* (**B**).

Therefore, stability experiments were conducted using the PBSE-modified biosensor in the second method. It was stored in 1 mM PB at 4 °C and evaluated for stability over 0, 3, 7, and 10 days. As shown in Figure 6, the biosensor retained its signal at 100% on the 3rd day and 75% on the 7th day of the study. After 10 days, the biosensor’s signal was 109%. These data show that the modified biosensor was stable for 10 days.

### 3.6. Validating the Sensitivity of the Biosensor with a Synthetic Serum Sample

**Figure 7:**
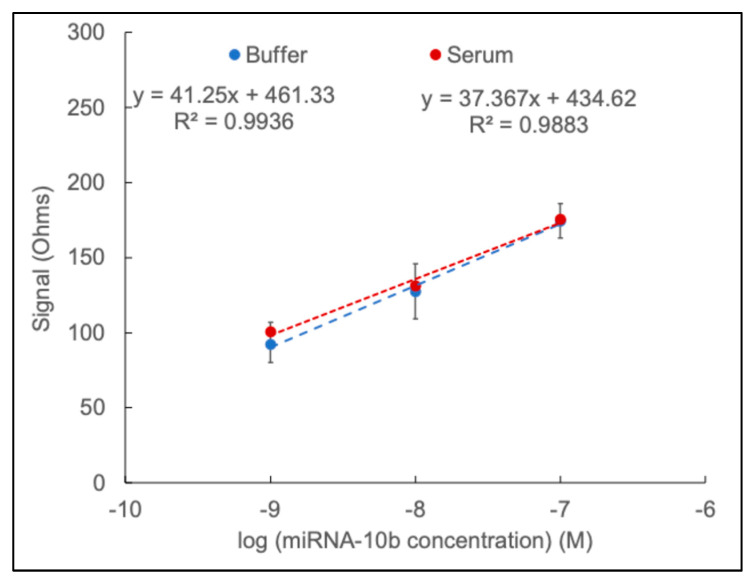
The calibration curve is based on the expected values of the buffer (black) and the actual values of the serum (red). The standard deviation bars are for the buffer.

The standard method was applied to buffer and serum samples to evaluate the potential application of the proposed biosensor. For this experiment, a two-fold diluted serum was incubated on the surface for one hour to reduce the matrix effect. Different concentrations of synthetic samples were incubated directly with the constructed interface. Table 1 shows the responses to concentrations ranging from 10^−7^ M to 10^−9^ M for both buffer and serum, and Figure 7 shows the calibration curve of the expected values based on the buffer and the actual values based on the serum. The results demonstrate that the actual serum values are closely related to the expected buffer values. This indicates that the biosensor has the ability to accurately detect the low concentration of the target analyte not only in buffer but also in serum. Therefore, the detection limit of the designed sensor in synthetic samples is 1 nM based on the EIS method. 

## 4. Conclusions

An electrochemical biosensor for detecting the breast cancer biomarker miRNA-10b was constructed on a glassy carbon electrode. The fabricated biosensor relies on the electrochemical signals generated by a hybridization reaction between the probe DNA and miRNA-10b. Graphene and PBSE binding together serves as great immobilization support, proving effective in binding the DNA molecules to the surface. Then, the target was exposed to the probe, and EIS was utilized to analyze the effectiveness of the developed biosensor. The LOD was observed as low as 10 pM. In addition, this biosensor exhibited strong specificity, enabling it to differentiate the miRNA target from other interfering oligonucleotides. The results suggest that the biosensor has immense potential for detecting breast cancer biomarkers in serum and can be applied for testing real clinical samples. 

The developed novel biosensor has excellent potential for sensitive and reliable TNBC biomarker detection. However, future directions could involve the simultaneous detection of multiple breast cancer biomarkers to provide a comprehensive diagnostic profile for improved decision-making. Additionally, the sensitivity of the biosensor could be explored by reducing the non-specific binding and background noise to achieve lower limits for early disease detection. The developed biosensor could be adapted to a screen-printed electrode as portable and versatile technology, creating a cost-effective, rapid diagnostic system for point-of-care testing. Such endeavors could be expanded further to propel the biosensor’s capabilities in revalorizing TNBC detection, offering new pathways for early, quick, personalized patient treatment strategies.

## Figures and Tables

**Table 1:** Expected values (buffer) compared to actual values (synthetic serum).

miRNA10b Concentration	Expected Median	Expected Range	Actual Median	Actual Range
**10^−7^ M**	174	163–186	175	125–265
**10^−8^ M**	128	109–146	131	90–166
**10^−9^ M**	92	80–107	78.5	76–81

## Data Availability

The authors confirm that the data supporting the findings of this study are available within the article and its Supplementary Materials. The data that support the findings of this study are available from the corresponding author, Ramaraja Ramasamy, upon reasonable request.

## Supplementary Materials

The following supporting information can be downloaded at: https://www.mdpi.com/article/10.3390/s24175747/s1, Figure S1: Time stability study of DNA-immobilized biosensor over 10 days.
