# Supplementary material for: An Electrochemical Nucleic Acid Biosensor for Triple-Negative Breast Cancer Biomarker Detection
Source: Sensors (Basel). 2024 Sep 4;24(17):5747. doi: 10.3390/s24175747 (PMC11397751; doi:10.3390/s24175747)
Supplement: Supplementary file 1 [file sensors-24-05747-s001.zip › sensors-3176175-SI.pdf]

Supplementary Section

DNA Immobilized Biosensor Stability

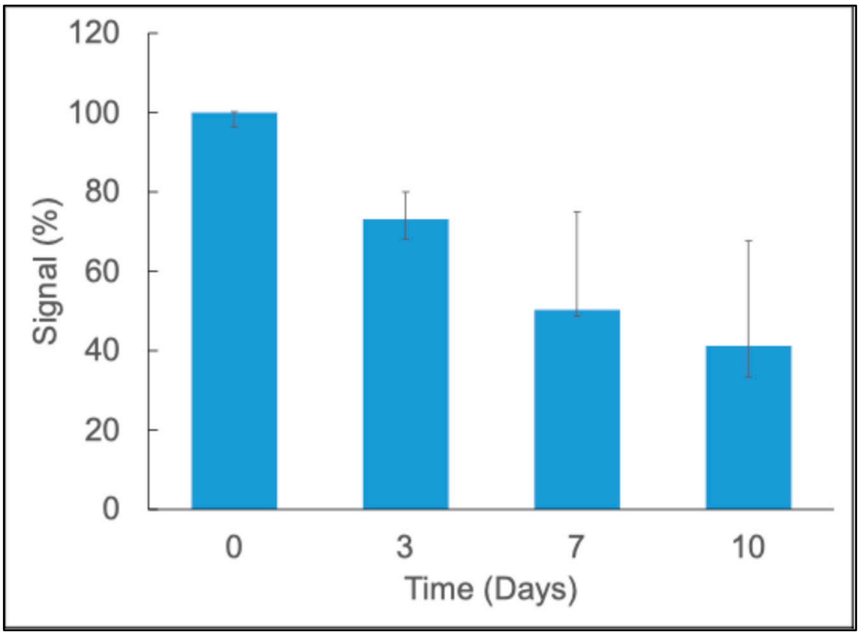

Figure S1. Time stability study of DNA immobilized biosensor over 10 days.
